# Individual Preparedness for Large-scale Earthquakes among International Students in Japan: A Cross-sectional Questionnaire Survey

**DOI:** 10.31662/jmaj.2024-0049

**Published:** 2024-09-06

**Authors:** Risa Koike, Nao Sonoda, Hideaki Furuki, Akiko Morimoto

**Affiliations:** 1 Graduate School of Nursing, Osaka Metropolitan University, Osaka, Japan

**Keywords:** preparedness, earthquake, international student, Japan

## Abstract

**Introduction:**

Individual preparedness for large-scale earthquakes is essential for safety and security in Japan, where earthquakes frequently occur. Foreign residents in Japan face barriers to gathering disaster information, and international students are likely to be more vulnerable to the effects of earthquakes due to the shorter duration of their stay in Japan. However, no studies have been conducted on international students’ individual preparedness for large-scale earthquakes in Japan. This study aimed to investigate individual preparedness for large-scale earthquakes among international students in Japan.

**Methods:**

This cross-sectional study was conducted from May to August 2023 among 360 international students aged ≥20 years enrolled at seven Japanese-language educational institutions in Osaka, Kyoto, and Hyogo prefectures. Of these, 120 (33.3%) agreed to participate in the mail surveys. Students with invalid answers were excluded and 114 (31.7%) were included in the analysis. The information obtained using a self-administered questionnaire included participants’ characteristics, methods used to collect information on individual preparedness for large-scale earthquakes (information sources and languages), and individual preparedness for large-scale earthquakes.

**Results:**

Many international students had not implemented safety measures at home and lacked information about safety confirmation, evacuation sites and routes, items to wear during evacuation, items to take in case of evacuation, or items to stockpile at home. In particular, approximately half of the participants lacked knowledge about nearby evacuation sites, and only 37.7% had confirmed their evacuation routes to nearby evacuation sites. Only 32.5% had prepared bags containing emergency items to take in case of an evacuation, and most had not packed the items, even though they were stocked at home. In addition, only 8.8% had stockpiled radios and emergency portable toilets at home.

**Conclusions:**

It is necessary to promote individual preparedness for large-scale earthquakes among international students in Japan.

## Introduction

Although Japan covers only 0.25% of the land area on the planet, approximately one-fifth of the world’s earthquakes, of magnitude 6.0 or greater, occur in Japan ^(1), (2), (3)^. Japan has approximately 2,000 active fault lines and is located on the boundaries of four plates (the Pacific Plate, Philippine Sea Plate, Eurasian Plate, and North American Plate) ^(4)^; thus, large-scale earthquakes can occur anywhere at any time. More than 6,400 people died from the Great Hanshin-Awaji Earthquake in 1995, and more than 18,000 people died or went missing due to the Great East Japan Earthquake in 2011 ^(1)^. There is a high probability of large-scale earthquakes in the near future, including impending possibilities of the Nankai Trough Earthquake and Tokyo Inland Earthquake ^(5), (6)^. Therefore, individual preparedness for large-scale earthquakes is necessary for safety and security in Japan, where earthquakes frequently occur.

Disaster reduction education is important to promote individual preparedness for large-scale earthquakes ^(1)^. In Japan, the government has designated the 1st day of September as the Disaster Preparedness Day and the week including this day as the Disaster Preparedness Week and promotes individual preparedness through disaster prevention drills and lectures ^(7)^. In addition, the Ministry of Education, Culture, Sports, Science and Technology has released a “Guide to Make a Disaster Reduction Manual for Schools (Earthquake and Tsunami)” and “Development of a Disaster Reduction Education to Nurture Power to Live On” to enhance disaster education at schools ^(8), (9)^. Moreover, the Fire and Disaster Management Agency has released a textbook for school teachers called “Challenge! Disaster Reduction 48” ^(10)^. Thus, Japanese people receive education about disaster reduction according to their stage of development, starting from early childhood.

Meanwhile, Japan has 3.22 million foreign residents ^(11)^. Individual risk from disasters is defined by disaster hazard (the disaster itself) and vulnerability, which is susceptibility to the effects of disasters when they occur (risk = hazard × vulnerability) ^(12)^. Foreign residents in Japan are considered more vulnerable to serious disasters because of limited information due to language barriers and unfamiliarity with natural hazard ^(13)^. In particular, more than 300,000 ^(11)^ international students in Japan have a shorter duration of stay. It has been reported that foreign residents with a shorter duration of stay in Japan are less familiar with their area of residence and have less knowledge about disaster reduction ^(14)^. Thus, international students in Japan are considered more vulnerable to the effects of earthquakes. Understanding the actual state of individual preparedness for large-scale earthquakes among international students in Japan would be useful in determining appropriate interventions to promote their preparedness. However, no studies on international students’ individual preparedness for large-scale earthquakes in Japan have been conducted. Therefore, this study aimed to investigate international students’ individual preparedness for large-scale earthquakes in Japan.

## Materials and Methods

### Study participants

After arriving in Japan, most international students first enroll in Japanese-language educational institutions ^(15)^. This cross-sectional study was conducted from May to August 2023 among international students aged ≥20 years enrolled at seven Japanese-language educational institutions in Osaka, Kyoto, and Hyogo prefectures, selected for convenience. The inclusion criteria were international students who had the N1 (the ability to understand Japanese used in various circumstances), N2 (the ability to understand Japanese used in everyday situations and in various circumstances to a certain degree), or N3 (the ability to understand Japanese used in everyday situations to a certain degree) of the Japanese-Language Proficiency Test ^(16)^ or those judged by teachers at Japanese-language educational institutions to have N3-equivalent Japanese-language proficiency. The exclusion criteria were international students who did not have the N1, N2, or N3 of the Japanese-Language Proficiency Test and those judged by teachers at Japanese-language educational institutions not to have N3-equivalent Japanese-language proficiency.

The required sample size was 97, calculated with a population size of 300,000 ^(11)^, a confidence level of 95%, and a margin of error of 10% ^(17)^. The response rate was assumed to be approximately 30%. A total of 360 international students were involved in this study, 120 of whom (33.3%) agreed to participate in the mail survey. After excluding those with invalid answers, 114 students (31.7%) were included in the analysis.

The study protocol was prepared in accordance with the Declaration of Helsinki and was approved by the institutional review board of Osaka Metropolitan University (approval date: March 24, 2023; approval no. 2023-30). Informed consent was obtained from all the participants included in this study.

### Data collection and questionnaires

Information obtained using the self-administered questionnaire were age, sex, nationality, Japanese-language proficiency, duration of stay in Japan, living status, economic status, experience participating in disaster prevention drills, sources of individual preparedness for large-scale earthquakes, and languages used when gathering information on individual preparedness for large-scale earthquakes.

In addition, we investigated the actual state of individual preparedness for large-scale earthquakes. Regarding necessary individual preparedness for large-scale earthquakes, we conducted a literature review. We searched published articles in electronic databases, PubMed and ICHUSHI (for Japanese articles), in May 2022. The search period was from January 2000 to April 2022. We used the following combinations of keywords: preparedness, disaster prevention, natural disaster, and earthquake. We also manually searched the reference lists of the identified articles and Google Scholar. Eligibility criteria were defined before the database search to ensure that only studies relevant to the research question were included. Inclusion criteria were as follows: (a) articles published in journals, (b) articles written in English or Japanese, (c) quantitative studies, and (d) articles that reported on necessary individual preparedness for large-scale earthquakes. The initial database search yielded 1,514 articles. Five articles were obtained via manual search. After screening, we identified 18 articles ^(18), (19), (20), (21), (22), (23), (24), (25), (26), (27), (28), (29), (30), (31), (32), (33), (34), (35)^. In addition, we searched Japanese public institutions’ websites and books regarding necessary individual preparedness for large-scale earthquakes and identified seven websites and books ^(36), (37), (38), (39), (40), (41), (42)^. Based on these literatures, we created detailed items on individual preparedness for large-scale earthquakes (Supplementary Appendix 1). In addition, we classified individual preparedness for large-scale earthquakes into four categories: (1) preparedness for home safety, (2) preparedness for safety confirmation and evacuation, (3) preparedness regarding items to take in case of evacuation, and (4) preparedness regarding items to stockpile at home. The presence or absence of preparedness was assessed for each item. The overall Kuder-Richardson coefficient was 0.917. The Kuder-Richardson coefficients of (1) preparedness for home safety, (2) preparedness for safety confirmation and evacuation, (3) preparedness regarding items to take in case of evacuation, and (4) preparedness regarding items to stockpile at home were 0.737, 0.740, 0.928, and 0.866, respectively.

Japanese-language expressions (grammar, sentence breaks, vocabulary, etc.) in the self-administered questionnaire were based on several guidelines and example sentences related to Easy Japanese (*Yasashii Nihongo*), a simplified version of written Japanese ^(43), (44), (45), (46), (47), (48)^. Illustrations were also included in the self-administered questionnaire.

### Statistical analyses

All variables were calculated using descriptive statistics. Age is shown as the median (25^th^ and 75^th^ percentiles). Dichotomous and categorical data are presented as numbers (%). All statistical analyses were performed using SPSS statistical software version 26 (IBM SPSS Japan, Tokyo, Japan).

## Results

### Participants’ characteristics

Table 1 shows the characteristics of the 114 international students. The median participant age was 23.0 years, and approximately half of the participants were men. More than 90% of participants were from Asian countries, with the largest number from China (34.2%), followed by Vietnam (23.7%) and Myanmar (20.2%). Moreover, 38.6% of the participants had an N3 level of Japanese-language proficiency, followed by those with N3-equivalent level (28.1%). More than 40% had stayed in Japan for less than 1 year, and approximately 50% had stayed in Japan for more than 1 year but less than 1 year and 6 months. More than 60% of participants lived alone, and none lived with Japanese people. Approximately 50% had never participated in disaster prevention drills, and only 15% had participated in disaster prevention drills in Japan, which is very low.

**Table 1.** Participants’ Characteristics and Methods Used to Collect Information on Individual Preparedness for Large-Scale Earthquakes.

	International students (n = 114)
Age (years)	23.0 (21.0, 25.0)
Men	53 (46.5)
Nationality: Asian countries	107 (94.1)
Japanese language proficiency*	
N1	10 (8.8)
N2	28 (24.6)
N3	44 (38.6)
N3-equivalent	32 (28.1)
Duration of stay in Japan	
Less than 6 months	17 (14.9)
More than 6 months but less than 1 year	32 (28.1)
More than 1 year but less than 1 year and 6 months	59 (51.8)
More than 1 year and 6 months	6 (5.2)
Living status	
Living alone	72 (63.2)
Living with non-Japanese	42 (36.8)
Living with Japanese	0 (0.0)
Economic status**	
Good	35 (30.7)
Average	49 (43.0)
Poor	29 (25.4)
Experience of participating in disaster prevention drills	
Participated in home country***	43 (37.7)
Participated in Japan***	19 (16.7)
Never participated	54 (47.4)
Information sources of individual preparedness for large-scale earthquakes***	
Websites	63 (55.3)
Social networking service	50 (43.9)
Newspapers	29 (25.4)
Television	29 (25.4)
Leaflets and booklets	13 (11.4)
Languages used when gathering information on individual preparedness for large-scale earthquakes***	
Native language	78 (68.4)
Japanese	74 (64.9)
English	41 (36.0)

Age was shown as median (25th, 75th percentiles). Dichotomous data and categorical data are shown as n (%). *N1, the ability to understand Japanese used in various circumstances; N2, the ability to understand Japanese used in everyday situations and in various circumstances to a certain degree; N3, the ability to understand Japanese used in everyday situations to a certain degree; and N3-equivalent, those judged by teachers at Japanese-language educational institutions as having N3-equivalent Japanese language proficiency. **n = 113 ***Multiple-choice answer

### Methods used by participants to collect information on individual preparedness for large-scale earthquakes

Table 1 shows the methods used by participants to collect information on individual preparedness for large-scale earthquakes. Websites (55.3%) was the most common information source for individual preparedness for large-scale earthquakes (multiple-choice answers), followed by social networking service (SNS) (43.9%). Approximately 25% used newspapers and television as information sources, and 11.4% obtained information from leaflets and booklets. Participants’ native language (68.4%) was the most common language used when gathering information on individual preparedness for large-scale earthquakes, followed by Japanese (64.9%) and English (36.0%).

### Actual situation of individual preparedness for large-scale earthquakes among international students in Japan

Table 2 shows participants’ preparedness for home safety. Many international students were not prepared for safety measures at home. Table 3 shows participants’ preparedness for safety confirmation and evacuation. Many international students were not prepared for safety confirmation and evacuation. Approximately half of them had not decided on how to confirm their safety with family and friends, and only approximately 20% knew how to use the Disaster Emergency Message Dial (171) and Disaster Emergency Message Board (Web171). Approximately half of the participants had not confirmed nearby evacuation sites, and only approximately 40% had confirmed their evacuation routes to the nearby evacuation sites. Only approximately 30% of participants had prepared bags containing emergency items to take in case of evacuation. Table 4 shows participants’ preparedness of items to take in case of evacuation. Most international students were not prepared with the items needed in case of evacuation. Table 5 shows participants’ preparedness regarding items to stockpile at home. Radios and emergency portable toilets were items not specifically stockpiled; only approximately 10% had stockpiled these items. Approximately half of the participants did not stockpile drinking water, and only approximately 40% had stockpiled emergency or preserved food. Approximately half of the participants did not stockpile regular medicines and medicines for chronic diseases, and only approximately 35% had stockpiled a first aid kit.

**Table 2.** Preparedness for Home Safety among the Participants.

	International students who answered “I am prepared”
Measures to prevent furniture and home appliances from falling due to earthquakes	
I attach fall prevention devices to the sides and tops of furniture and home appliances	14 (12.3)
I place sticky mats, stoppers, etc. under furniture and home appliances	20 (17.5)
I store heavy items in the bottom of storage furniture (shelves, etc.)	32 (28.1)
I do not place furniture or home appliances near beds or futons	50 (43.9)
I do not place furniture or home appliances near the entrance to my room	54 (47.4)
Measures to prevent objects from falling due to earthquakes	
I put anti-opening devices on doors and drawers of storage furniture	7 (6.1)
I do not place objects on top of furniture or home appliances	34 (29.8)
Measures to prevent glass breakage due to earthquakes	
I put shatterproof film on glass (windows, cupboard doors, etc.)	9 (7.9)
Measures to prevent fires caused by earthquakes
I know how to use the nearby fire extinguisher	31 (27.2)
I know how to turn off the breaker	47 (41.2)
I know the location of the breaker	51 (44.7)
I know the location of nearby fire extinguishers	62 (54.4)

Dichotomous data are shown as n (%). In each category, items are listed in ascending order of percentage.

**Table 3.** Preparedness for Safety Confirmation and Evacuation among the Participants.

	International students who answered “I am prepared”
Preparedness for safety confirmation	
I know how to use the Disaster Emergency Message Dial (171) and Disaster Emergency Message Board (Web171)	27 (23.7)
I have decided how to confirm my safety with family and friends	53 (46.5)
Preparedness for evacuation sites and routes
I know the evacuation routes to the nearby evacuation sites	43 (37.7)
I know nearby evacuation sites	61 (53.5)
Items prepared at home (current residence) to wear during evacuation	
Whistle	11 (9.6)
Helmet	20 (17.5)
Gloves	26 (22.8)
Light (headlight, neck light, flashlight, etc.)	31 (27.2)
Bag containing emergency items	37 (32.5)
Long-sleeve shirt and long pants	61 (53.5)
Sneakers	72 (63.2)

Dichotomous data are shown as n (%). In each category, items are listed in ascending order of percentage.

**Table 4.** Preparedness Regarding Items to Take in Case of Evacuation among the Participants.

	International students who answered “I am prepared”
Valuables	
Copy of cash card and bankbook	16 (14.0)
Copy of identification documents (passport, residence card)	22 (19.3)
Cash	32 (28.1)
Information equipment	
Radio	1 (0.9)
Mobile battery for smartphone and mobile phone	26 (22.8)
Drinking water and emergency food	
Disposable tableware (disposable chopsticks, paper cups, paper plates, etc.)	5 (4.4)
Emergency food and preserved food (instant food, etc.)	9 (7.9)
Drinking water	13 (11.4)
Medical and hygiene supplies	
Toothbrush and toothpaste	4 (3.5)
Hand rubbing alcohol	7 (6.1)
First aid kit (plasters, bandages, disinfectant, thermometer, etc.)	8 (7.0)
Sanitary items*	7 (11.9)
Regular medicine and medicine for chronic diseases	14 (12.3)
Mask	17 (14.9)
Daily necessities	
Emergency portable toilet	6 (5.3)
Toilet paper	7 (6.1)
Tissue paper	7 (6.1)
Large plastic bag	8 (7.0)
Towel and large handkerchief	8 (7.0)
Change of clothes	9 (7.9)
Cold weather gear (warmer, aluminum blanket, etc.)	10 (8.8)
Rain gear (raincoat, umbrella, etc.)	14 (12.3)

Dichotomous data are shown as n (%). In each category, items are listed in ascending order of percentage. *Women only (n = 59)

**Table 5.** Preparedness Regarding Items to Stockpile at Home among the Participants.

	International students who answered “I am prepared”
Valuables	
Copy of cash card and bankbook	36 (31.6)
Copy of identification documents (passport, residence card)	62 (54.4)
Cash	70 (61.4)
Information equipment	
Radio	10 (8.8)
Mobile battery for smartphone and mobile phone	71 (62.3)
Drinking water and emergency food	
Disposable tableware (disposable chopsticks, paper cups, paper plates, etc.)	28 (24.6)
Emergency food and preserved food (instant food, etc.)	46 (40.4)
Drinking water	64 (56.1)
Medical and hygiene supplies	
Hand rubbing alcohol	34 (29.8)
First aid kit (plasters, bandages, disinfectant, thermometer, etc.)	40 (35.1)
Toothbrush and toothpaste	59 (51.8)
Regular medicine and medicine for chronic diseases	60 (52.6)
Mask	69 (60.5)
Sanitary items*	40 (67.8)
Daily necessities	
Emergency portable toilet	10 (8.8)
Cold weather gear (warmer, aluminum blanket, etc.)	37 (32.5)
Large plastic bag	45 (39.5)
Towel and large handkerchief	47 (41.2)
Toilet paper	53 (46.5)
Tissue paper	54 (47.4)
Change of clothes	57 (50.0)
Rain gear (raincoat, umbrella, etc.)	67 (58.8)

Dichotomous data are shown as n (%). In each category, items are listed in ascending order of percentage. *Women only (n = 59)

## Discussion

To our knowledge, this study is the first to focus on international students’ preparedness for large-scale earthquakes in Japan. This study showed that many international students in Japan were not prepared for large-scale earthquakes.

In particular, this study showed that many international students did not prepare for their safety during an earthquake or evacuation. During the 1995 Great Hanshin-Awaji Earthquake, approximately 200,000 houses were damaged, and approximately 90% of deaths resulted from being crushed and suffocated in collapsed houses ^(49)^. Furthermore, approximately 400,000 houses were damaged in the 2011 Great East Japan Earthquake. Although earthquake countermeasures for buildings, such as seismic retrofitting, have progressed in recent years, it is estimated that approximately 2.08 million houses will be damaged in the Nankai Trough Earthquake and approximately 850,000 houses in the Tokyo Inland Earthquake ^(50), (51)^. Therefore, although individual preparedness for safety during an earthquake or evacuation is important, most participants had not implemented safety measures at home. In addition, most participants lacked the requisite items to be worn during an evacuation. A recent large-scale study involving community residents in Osaka Prefecture reported that approximately 30% of them had fixed their furniture for home safety ^(33)^. In addition, a recent study involving university students living alone at 10 universities in Japan reported that approximately 30% of them had fixed their furniture for home safety ^(28)^. However, approximately 10% of international students in this study answered “I attach fall prevention devices to the sides and tops of furniture and home appliances,” indicating that international students are less prepared. Although this study did not investigate the reasons for not preparing for large-scale earthquakes, a previous study reported that foreign residents unfamiliar with earthquakes do not understand the strength of a seismic intensity 7 earthquake ^(52)^. Moreover, approximately 50% of international students in this study had never participated in disaster prevention drills, and only approximately 15% had participated in disaster prevention drills in Japan, which is very low. Therefore, disaster reduction education, including disaster prevention drills, for international students is necessary.

In addition, preparedness regarding safety confirmation, evacuation sites, and routes was also inadequate. In this study, more than 40% of participants had lived in Japan for less than 1 year, and approximately 50% had lived in Japan for more than 1 year, but less than 1 year and 6 months. Moreover, more than 60% of participants lived alone, and none lived with Japanese people. It is estimated that the Nankai Trough Earthquake will cause 5.8 million communication failures ^(50)^. Thus, little evacuation information would be available to international students in their native languages in case of an earthquake. Moreover, not knowing nearby evacuation sites or routes will affect the safety of international students. Furthermore, international students would feel more anxious if they are unable to contact their family and friends due to communication failures. After arriving in Japan, most international students first enroll in Japanese-language educational institutions ^(15)^. Therefore, it would be important to conduct disaster reduction education, such as safety measures, safety confirmation, and evacuation, at the time of enrollment in these institutions.

This study also showed that many international students in Japan were not prepared with items to take with them in the case of evacuation or items to stockpile at home. In addition to the communication failures described above, it was estimated that the Nankai Trough Earthquake could cause power outages in approximately 29 million houses, gas outages in approximately 1.8 million houses, and water outages in approximately 36 million houses^(50)^. Furthermore, in the event of a large earthquake, it takes a long time to restore lifeline outages ^(50)^. The recent study involving university students living alone at 10 universities in Japan reported that approximately 70% of them were prepared with items to take during an evacuation ^(28)^. In this study, only approximately 30% of international students had prepared bags containing emergency items to take during an evacuation, and most had not packed the items, even though they were stocked at home. Moreover, few international students had stockpiled radios and emergency portable toilets at home. Since the most common information source of information for individual preparedness for large-scale earthquakes was websites (55.3%), followed by SNS (43.9%), it would be important to establish e-learning programs on individual preparedness for international students. The Fire and Disaster Management Agency offers an internet program called “Disaster Reduction/Crisis Management e-College” directed toward children and community residents ^(53)^. It would be necessary to provide such e-learning programs in multiple languages to promote individual preparedness among international students.

This study had some limitations. First, there is a possibility of selection bias. There are more than 2,000 Japanese-language educational institutions in Japan, where approximately 160,000 international students are enrolled ^(54)^. This cross-sectional study was conducted with international students affiliated with seven Japanese-language educational institutions in the Osaka, Kyoto, and Hyogo prefectures. In addition, the inclusion criteria were international students who had the N1, N2, or N3 of the Japanese-Language Proficiency Test ^(16)^ or those judged by teachers at Japanese-language educational institutions to have N3-equivalent Japanese-language proficiency. Furthermore, the response rate for this study was 33.3%, suggesting that those who were more concerned about earthquakes and were relatively prepared may have been selected. Second, although the Japanese-language expressions (grammar, sentence breaks, vocabulary, etc.) in the self-administered questionnaire were based on several guidelines and example sentences related to Easy Japanese (*Yasashii Nihongo*), a simplified version of written Japanese ^(43), (44), (45), (46), (47), (48)^, and illustrations were also included in the self-administered questionnaires, there is a possibility of information bias in data collection. Third, the test-retest reliability and validity were not examined for the items on individual preparedness for large-scale earthquakes. Finally, to simplify the self-administered questionnaire, we did not collect data on the content of disaster prevention drills.

In conclusion, this study showed that several international students had not implemented safety measures at home and lacked information about safety confirmation, evacuation sites and routes, items to wear during evacuation, items to take in case of evacuation, and items to stockpile at home. Disaster reduction education at Japanese-language educational institutions and e-learning programs would be important to promote individual preparedness among international students. Further research on international students’ knowledge of earthquakes and disaster reduction, the content of disaster reduction education received in home countries, and the actual situation of disaster reduction education at Japanese-language educational institutions are needed.

## Article Information

### Conflicts of Interest

None

### Sources of Funding

This work was supported by JSPS KAKENHI grant number JP 22K11120

### Acknowledgement

We thank all the participants who took part in this study.

### Author Contributions

RK: conceptualization, data collection, data analysis, interpretation of results, and writing of the original draft. HF: interpretation of results and review and editing. NS and AM: conceptualization, funding acquisition, interpretation of results, project administration, and review and editing. All authors reviewed and approved the final version of the manuscript.

### Approval by Institutional Review Board (IRB)

The study protocol was prepared in accordance with the Declaration of Helsinki and approved by the Institutional Review Board of Osaka Metropolitan University (approval date: March 24, 2023; approval no. 2023-30).

### Informed Consent

Informed consent was obtained from all participants in this study.

### Data Availability

Data cannot be shared for privacy or ethical reasons.

## Supplement

Supplementary Appendix 1
