# Supplementary material for: Individual Preparedness for Large-scale Earthquakes among International Students in Japan: A Cross-sectional Questionnaire Survey
Source: JMA J. 2024 Sep 6;7(4):496–505. doi: 10.31662/jmaj.2024-0049 (PMC11543362; doi:10.31662/jmaj.2024-0049)
Supplement: Supplementary Appendix 1 [file 2433-3298-7-4-0496-s001.pdf]

Supplementary Appendix 1. Self-administered questionnaire on individual preparedness for large-scale earthquakes

Preparedness for home safety

Please check (☑) if you take safety measures at home (current residence) to prepare for earthquakes.
Please do not check if you are not prepared.

	Please check (☑) if you take safety measures at home
1) I place sticky mats, stoppers, etc[※1]. under furniture and home appliances	☐
2) I attach fall prevention devices[※2] to the sides and tops of furniture and home appliances	☐
3) I store heavy items in the bottom of storage furniture (shelves, etc.)	☐
4) I do not place furniture or home appliances near the entrance to my room	☐
5) I do not place furniture or home appliances near beds or futons	☐
6) I put anti-opening devices[※3] on doors and drawers of storage furniture	☐
7) I do not place objects on top of furniture or home appliances	☐
8) I put shatterproof film[※4] on glass (windows, cupboard doors, etc.)	☐
9) I know the location of the breaker	☐
10) I know how to turn off the breaker	☐
11) I know the location of nearby fire extinguishers	☐
12) I know how to use the nearby fire extinguisher	☐

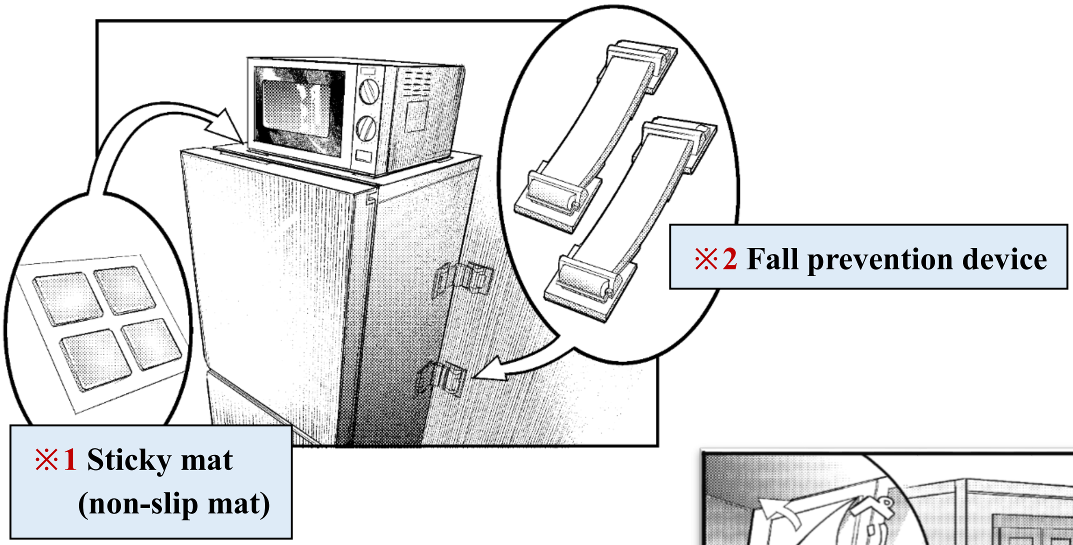

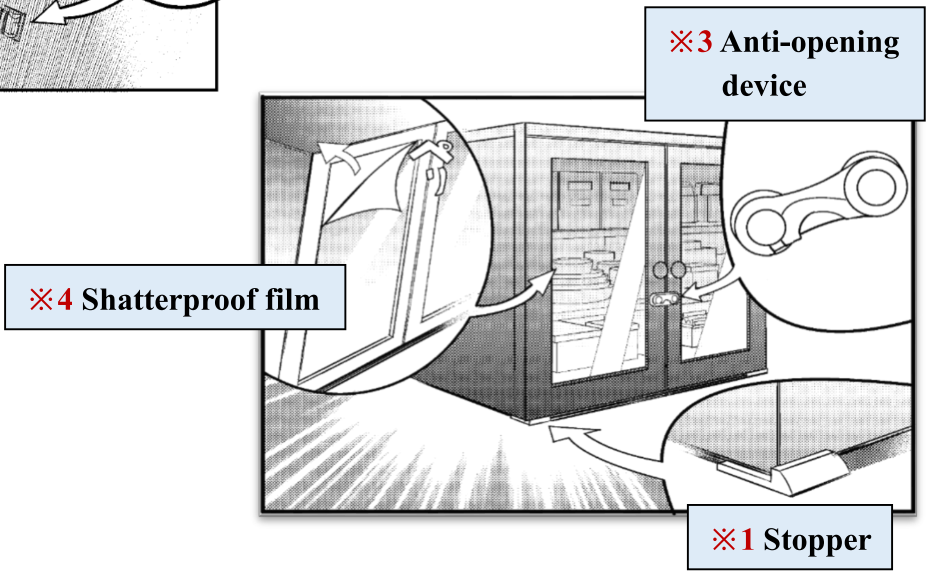

Preparedness for safety confirmation and evacuation

Please check (☑) if you are prepared for earthquakes.
Please do not check if you are not prepared.

	Please check (☑) if you are prepared for earthquakes
1) I have decided how to confirm my safety with family and friends	☐
2) I know how to use the Disaster Emergency Message Dial (171) and Disaster Emergency Message Board (Web171)	☐
3) I know nearby evacuation sites	☐
4) I know the evacuation routes to the nearby evacuation sites	☐

Please check (☑) if you are prepared items at home (current residence) to wear during evacuation.
Please do not check if you are not prepared.

	Please check (☑) if you are prepared items at home to wear during evacuation
1) Helmet	☐
2) Long sleeves shirt and long pant	☐
3) Gloves	☐
4) Sneakers	☐
5) Light (headlight, neck light, flashlight, etc.)	☐
6) Whistle	☐
7) Bag containing emergency items	☐

Preparedness regarding items to take in case of evacuation

Preparedness regarding items to stockpile at home

Please check (☑) in column A if you stockpile items at home (current residence).

Please also check (☑) in column B if you stockpile items in your evacuation bag at home (current residence).

Please do not check if you are not prepared.

	[A] Please check (☑) if you stockpile items at home	[B] Please also check (☑) if you stockpile items in your evacuation bag at home
1) Cash	☐	☐
2) Copy of identification documents (passport, residence card)	☐	☐
3) Copy of cash card and bankbook	☐	☐
4) Mobile battery for smartphone and mobile phone	☐	☐
5) Radio	☐	☐
6) Drinking water	☐	☐
7) Emergency food and preserved food (instant food, etc.)	☐	☐
8) Disposable tableware (disposable chopsticks, paper cups, paper plates, etc.)	☐	☐
9) Regular medicine and medicine for chronic diseases	☐	☐
10) First aid kit (plasters, bandages, disinfectant, thermometer, etc.)	☐	☐
11) Hand rubbing alcohol	☐	☐
12) Mask	☐	☐
13) Toothbrush and toothpaste	☐	☐
14) Sanitary items ※women only	☐	☐
15) Emergency portable toilet	☐	☐
16) Toilet paper	☐	☐
17) Tissue paper	☐	☐
18) Large plastic bag	☐	☐
19) Towel and large handkerchief	☐	☐
20) Change of clothes	☐	☐
21) Cold weather gear (warmer, aluminum blanket, etc.)	☐	☐
22) Rain gear (raincoat, umbrella, etc.)	☐	☐
